# Editorial: Host–guest chemistry of macrocycles— Volume II

**DOI:** 10.3389/fchem.2023.1162019

**Published:** 2023-02-21

**Authors:** Tangxin Xiao, Robert Elmes, Yong Yao

**Affiliations:** ^1^ School of Petrochemical Engineering, Changzhou University, Changzhou, China; ^2^ Department of Chemistry, Maynooth University, National University of Ireland, Maynooth, Ireland; ^3^ Synthesis and Solid-State Pharmaceutical Centre (SSPC), Maynooth University, National University of Ireland, Maynooth, Ireland; ^4^ School of Chemistry and Chemical Engineering, Nantong University, Nantong, China

**Keywords:** host-guest chemistry, macrocyclic molecules, supramolecular self-assembly, pillar[n]arenes, cucurbit[n]urils

The field of macrocyclic host–guest chemistry was born with the discovery of crown ethers (CEs) by Pederson and has been the catalyst that led to the field of supramolecular chemistry more generally (Pedersen, 1967). Apart from CEs, the diversity of macrocyclic hosts with different shapes and cavities is staggering and many other examples such as cyclodextrins (CDs), (Szejtli, 1998; Harada, 2001; Davis and Brewster, 2004; Antoniuk and Amiel, 2016) cucurbit[n]urils (CB[n]s), (Lagona et al., 2005; Masson et al., 2012; Ni et al., 2014; Murray et al., 2017; Palma et al., 2017; Chen et al., 2022a) calix[n]arenes (CA[n]s), (Diamond and McKervey, 1996; Baldini et al., 2007; Kim and Quang, 2007; Guo and Liu, 2014) pillar[n]arenes (PA[n]s) (Ogoshi et al., 2008; Xue et al., 2012; Kakuta et al., 2018; Ogoshi et al., 2018; Xiao et al., 2018; Xiao et al., 2019a; Xiao et al., 2019b; Xiao et al., 2019c; Ma et al., 2022; Wang et al., 2022) and other macrocyclic molecules have been reported (Chen and Han, 2018; Wang et al., 2020a; Peng et al., 2020; Wu et al., 2022; Zhang and Li, 2022). The dynamic binding behaviour of many different hosts and guests endows these materials with useful properties including stimuli-responsiveness, self-healing, degradability and recyclability. As a result, host–guest chemistry plays an important role in many research areas, such as supramolecular polymers, (Harada et al., 2009; Xiao et al., 2020a; Wu and Xiao, 2020; Hua et al., 2022) supramolecular networks/frameworks, (Wang et al., 2018; Wang et al., 2020b; Huang et al., 2020; Li et al., 2021) drug delivery, (Duan et al., 2013; Braegelman and Webber, 2019) artificial light-harvesting systems, (Xiao et al., 2019d; Xiao et al., 2022; Xiao et al., 2023) and dynamic hydrogels (Xiao et al., 2019e; Chen et al., 2022b) etc. In this context, we organized the Research Topic “host–guest chemistry of macrocycles” in 2020 which led to the publication of 15 important articles demonstrating the latest research in this area (Xiao et al., 2020b). Because of the importance and popularity of this Research Topic, we have now organised a second volume on this topic. Herein, we briefly introduce the works in this new Research Topic.

The structure of molecules in the solution is determined by the intrinsic properties of the molecules and their interaction with the surrounding solvent. Therefore, the protonation site of a molecule depends on both their intrinsic strengths and different stabilizations by the solvent. The complexation of a guest molecule with multiple basic sites and a macrocyclic host could stabilize, probe, or facilitate the formation of a specific protomer. To verify this, Alcázar et al. investigated the impact of CB[7] on the synthetic dyes 7-(dialkylamino)-aza-coumarin derivatives (SACs), which bear two basic sites in their structure (Alcázar et al., 2022). They first synthesized three styryl-derived SACs, namely **SAC1**, **SAC2**, and **SAC3**, which were fully characterized by HR ESI-MS, IR, and NMR. The spectral behavior of the SACs in the absence and presence of CB7 was studied. In contrast to the heterocyclic nitrogen, the dialkylamino nitrogen in **SAC1** (pKa = 1.30) and **SAC2** (pKa = 2.35) are more likely to be protonated. However, the protonation of **SAC3** could take place both in the heterocyclic nitrogen (pKa = 1.67) and dialkylamino nitrogen (pKa = 1.75) independently. These protomers of the SACs were confirmed by UV-vis absorbance experiments and DFT calculations. Intriguingly, in the presence of CB[7], the heterocyclic nitrogen was favored to be protonated over the dialkylamino nitrogen, which may be due to a change in the protonation preference of SACs induced by CB[7] upon host–guest interaction. Notably, a bathochromic shift of ≈4500 cm^−1^ (**SAC1-3**) was observed in the presence of CB[7].

Fluorescent indicator displacement (FID) assays are an excellent method to probe analytes *via* the conversion of receptors into optical sensors that can sense binding of different guests. Given the rapid development of supramolecular host–guest chemistry, macrocycle-based FID assays have received considerable attention due to their potential in the area of chemical sensing. Food additives based on phenolic compound play a key role in the food industry on account of their remarkable antibacterial and antioxidant properties. However, the excessive use and accumulation of food additives in the environment is gradually increasing and is responsible for significant environmental concern. In this context, Duan et al. prepared a novel FID assay based on a cationic PA[6] (**CP6**) for the detection of some vital phenolic food additives, such as *p*-gallic acid (**GA**), trans-ferulic acid (**FA**) and coumaric acid (**CA**) (Duan et al., 2022a). The 6-*p*-toluidinylnaphthalene-2-sulfonate (**TNS**) was used as the fluorescent indicator in this FID system due to its enhanced emission in non-polar environments. Upon the host-guest complexation of **CP6** and **TNS**, the fluorescence of **TNS** was switched on. However, in the presence of **GA**, **FA** and **CA**, the fluorescence was turned off due to the guest’s displacement from the cavity of **CP6**. As a result, the host–guest chemistry-based FID system can be used as a sensor towards these phenolic food additives.

In a minireview paper, Duan et al. further summarized recent progresses on FID assays based on macrocyclic arenes (Duan et al., 2022b). The authors mainly divided the content to two parts: CA[n]-based FID assays and PA[n]-based FID assays. Due to their unique macrocyclic structure and versatile host–guest binding behaviors, the combination of CA[n]s or PA[n]s with various fluorophores is broadly used in FID assays for the specific and selective sensing of analytes. There is a larger diversity of reported analytes including neutral molecules, anions, cations, and biomolecules. Finally, the authors discussed the prospect and remaining challenges in this research area.

Hydrophobic interactions are another important non-covalent force in supramolecular chemistry. Nanoparticles (NPs) formed by surfactants in water are usually driven by hydrophobic interactions. In this Research Topic, Zhu et al. reported that multiple W/O/W (W: water, O: oil) emulsions can be obtained by using CaCO_3_ NPs and a surfactant (SDS) of different concentrations (Zhu et al., 2022). Once the low surface-activity CaCO_3_ NPs are treated, they become surface-active *in situ* with the addition of SDS. These emulsions possess very high stability, which can stand for at least 1 month without coalescence. The strategy provided in this work not only promotes stabilization of multiple emulsions but also avoids the tedious synthesis often associated with colloidal NPs.

In conclusion, this Research Topic collects some new advances in macrocycle-based host–guest chemistry covering a broad range of supramolecular chemistry research. As the field further develops, we believe host–guest chemistry will continue to be a source of intrigue for basic science and gradually, with investment and critical thinking provide real-world applications across health, environmental and materials science.
